# Case Report: Successful application of modified laparoscopic assisted percutaneous gastropexy in a dog using two 6-mm portal sites

**DOI:** 10.3389/fvets.2025.1614761

**Published:** 2025-11-20

**Authors:** Sohee Youn, Ho Hyun Kwak, Seung Jun Yoon, Junhyung Kim, Heung Myong Woo

**Affiliations:** 1 Department of Veterinary Surgery, College of Veterinary Medicine, Institute of Veterinary Science, Kangwon National University, Chuncheon, Republic of Korea; 2 Department of Companion Animal Industry, College of Natural and Life Sciences, Daegu University, Gyeongsan, Republic of Korea

**Keywords:** laparoscopy, prophylactic gastropexy, gastric dilatation and volvulus (GDV), laparoscopic gastropexy, barbed suture, minimal invasive surgery (MIS)

## Abstract

Gastric dilatation and volvulus (GDV) is a life-threatening disease in dogs and has a high rate of recurrence without gastropexy. However, prophylactic gastropexy effectively reduces the incidence of GDV. In a 5-year-old female Russo-European Laika, who had a high risk of GDV due to being purebred and deep-chested, and a positive family history, prophylactic gastropexy—utilizing two 6-mm ports and barbed sutures—was performed using the modified laparoscopic-assisted percutaneous gastropexy (mLAPG) technique, without open celiotomy and intracorporeal suturing. The gastropexy suturing time was 29 min. Follow-up assessments using ultrasonography, laparoscopy, and endoscopy were conducted 1 month postoperatively and confirmed stable adhesion without gastric wall damage or complications at the gastropexy site. This is the first case report of the application of mLAPG in a dog. Based on the successful formation of adhesion and the absence of complications for 1 year in this case, the mLAPG technique can be recommended as an effective method for prophylactic gastropexy in dogs.

## 1 Introduction

Gastric dilatation and volvulus (GDV), characterized by rapid twisting of the stomach along its mesenteric axis, most commonly in a clockwise direction, is a critical and life-threatening condition in dogs. Despite timely diagnosis, the recurrence rate of GDV can exceed 75% in the absence of surgical intervention (1, 2). Management of GDV without gastropexy frequently leads to a significantly shorter median survival time of 188 days, compared with 547 days in dogs that underwent gastropexy (3).

Prophylactic gastropexy greatly reduces the lifetime mortality associated with GDV (to 0.3%) and when GDV does occur, gastropexy significantly lowers the risk of recurrence (to <5%) (4, 5). Traditional open gastropexy procedures (6–12) require celiotomy, which can lead to postoperative pain and inflammatory reactions (13). To reduce surgical invasiveness, prophylactic gastropexy has also been performed using laparoscopic- or endoscopic-assisted methods (14–17), and total laparoscopic approaches (4, 18–24). The total laparoscopic gastropexy (TLG) has become increasingly popular owing to the associated low morbidity, quick recovery, and successful adhesion (4, 19, 20, 22, 24–27). The most challenging and time-consuming parts of these techniques are intracorporeal suturing and knot tying; however, to overcome this difficulty, several methods using barbed sutures and specialized laparoscopic suture devices have been developed (4, 20, 22–26, 28).

The percutaneous internal ring suturing technique was originally designed for inguinal hernia repair in children and requires only a single umbilical port, along with skin puncture using an 18-gauge injection needle. Under laparoscopic guidance, a needle with a nonabsorbable thread was inserted through the abdominal wall into the peritoneal cavity. By manipulating the needle, the thread was passed around the hernia ring, brought out through the abdominal wall again, tightened extracorporeally, and secured in the subcutaneous space (29). Iacona et al. adapted this technique, developing the laparoscopic-assisted percutaneous gastropexy (LAPG) for anterior gastropexy to manage acute and chronic gastric volvulus in infants (30). LAPG involves one additional instrumental port to manipulate the stomach and non-absorbable conventional suture material.

We applied a modified version of LAPG technique for prophylactic gastropexy in a dog. In comparison to previous LAPG (14), (1) the gastropexy site was adjusted to the right cranial abdomen due to the anatomical difference in the direction of gastric volvulus between human infants and dogs, and (2) barbed sutures without incision were used to induce permanent adhesion with higher tensile strength. This is the first case report describing the clinical feasibility of prophylactic gastropexy using the non-incisional mLAPG technique in a dog, in which gastropexy site adhesion was confirmed during postoperative follow-up.

## 2 Case description

A 5-year-old, 25-kg, female Russo-European Laika was presented to the Veterinary Medical Teaching Hospital of Kangwon National University for prophylactic gastropexy, with a history of her mother having died from GDV. The dog was bright, alert, responsive, and there were no remarkable findings on physical examination, thoracoabdominal radiography, or blood examination (complete blood count (CBC), serum chemistry, and electrolyte panel). The patient was premedicated with intravenously (IV) administered 0.3 mg/kg midazolam (Midazolam, Inj^®^; Bukwang Pharm, Korea) and 0.3 mg/kg butophanol (Butophan inj^®^, Myungmoon Pharm, Korea). Anesthesia was induced using 5 mg/kg propofol IV (Anepol inj^®^; Hana Pharm, Korea) and general anesthesia was maintained with isoflurane inhalation, via an endotracheal tube. Cefazoline 22 mg/kg (Cefazoline inj^®^; Chongkundang, Korea), a prophylactic antibiotic, was administered IV preoperatively.

**Figure 1:**
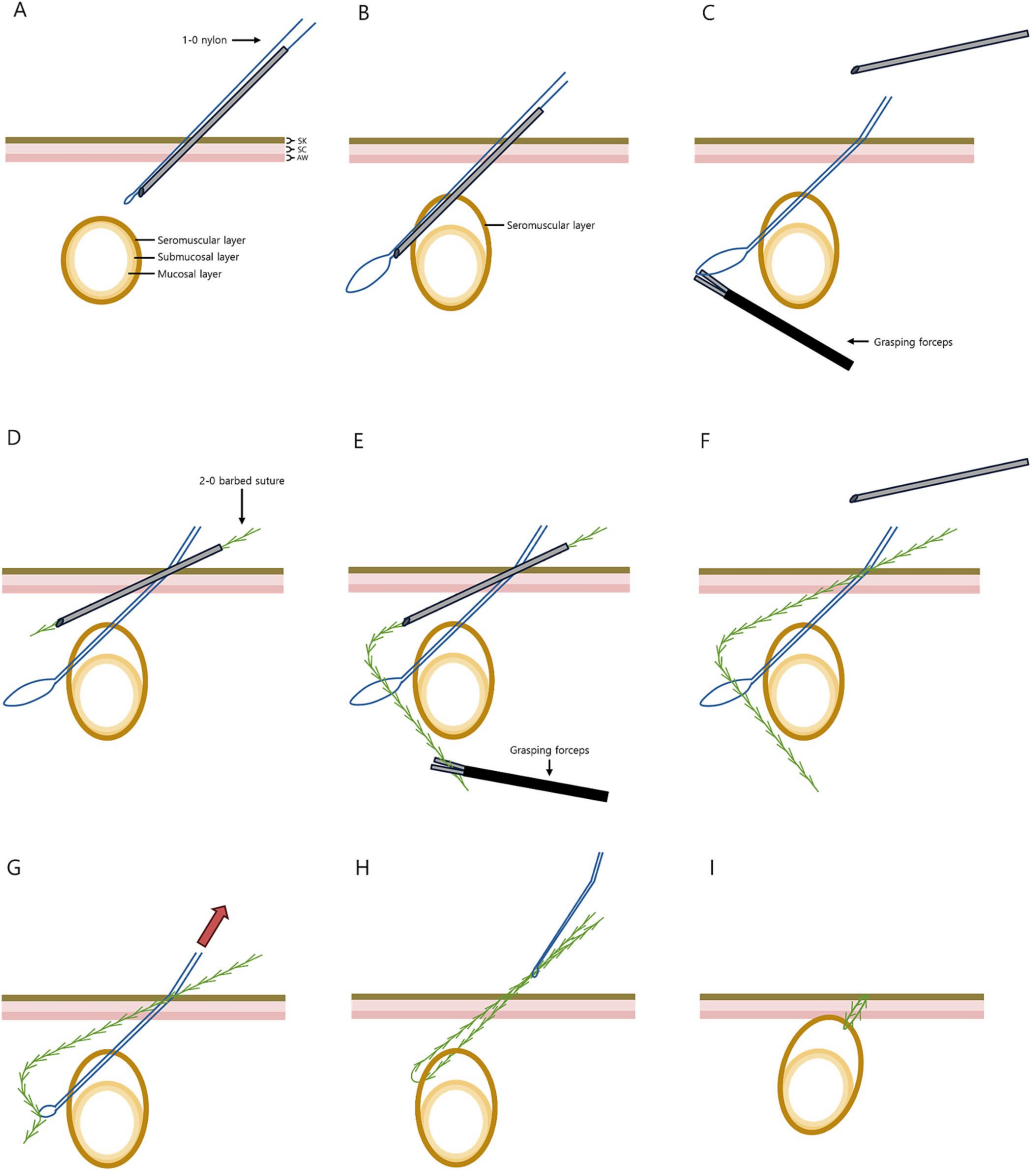
Illustrative overview of the modified laparoscopic-assisted percutaneous gastropexy (mLAPG) technique. SK, skin; SC, subcutaneous; AW, abdominal wall.

**Figure 2:**
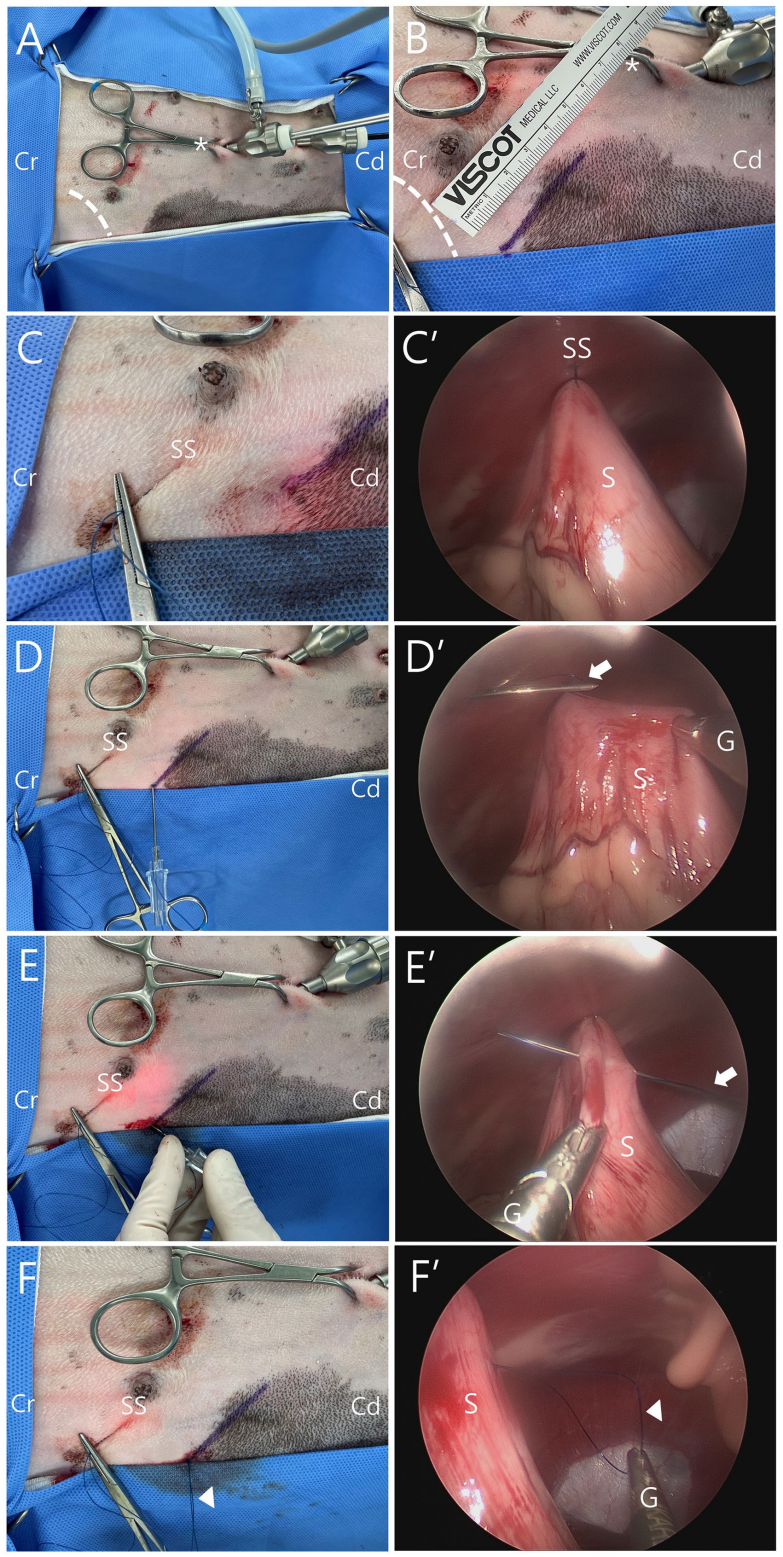
Intraoperative images of modified laparoscopic assisted percutaneous gastropexy (mLAPG) in a dog (X, X’: external and laparoscopic views at the same stage of the procedure). **(A)** An external image of laparoscopic portal placement. **(B)** The gastropexy site was marked in the right upper abdomen (solid purple line). **(C,C′)** Transabdominal stay suture (SS) using 1–0 nylon. The pyloric antrum is anchored to the abdominal wall. **(D,D′)** The needle of a 16-gauge intravenous catheter with a 1–0 nylon (arrow) creating a loop being introduced through the abdominal wall into the abdominal cavity. **(E,E’)** The needle of catheter with the nylon (arrow) passed through the seromuscular layer of the stomach. **(F,F′)** The catheter being removed, leaving the nylon (arrow head) inside the abdominal cavity. Cr, the cranial part of the patient; Cd, the caudal part of the patient; dotted line, 13th rib; asterisk, umbilicus; S, stomach; G, grasping forceps.

The mLAPG procedure using two 6-mm ports was performed in a dorsal recumbency (Figure 1). Using a Veress needle, the abdomen was insufflated with CO_2_ at a pressure of 10–12 mm Hg. The first cannula (Ternamian Endotip, Karl-Storz Veterinary Endoscopy, Goleta, CA) for telescope (5-mm, 0°, HOPKINS^®^ II Straight Forward Telescope, Karl-Storz Veterinary Endoscopy, Goleta, CA) was inserted 1 cm caudal to the umbilicus, and the second cannula for 5-mm laparoscopic fundus grasping forceps (Karl-Storz Veterinary Endoscopy, Goleta, CA) was inserted 3 cm caudal to the first one (Figure 2A). The pexy site was marked on the right upper abdomen with a 4-cm line, positioned 2–3 cm caudal and nearly perpendicular to the 13th rib on the right side, and 5–8 cm lateral to the ventral midline (Figure 2B). The avascular region of the pyloric antrum was grasped with fundus-grasping forceps and positioned to approximate the previously marked gastropexy site. A percutaneous stay suture was then placed transabdominally using 1-0 nylon, 2–3 cm cranial to the skin mark (Figures 2C,C’).

Before placing the first bite of the mLAPG, one end of a 1-0 nylon thread was passed through the barrel of a 16G, 45-mm intravenous catheter (BD Angiocath Plus; Becton-Dickinson and Company, Franklin Lakes, NJ, United States), with the other end folded back at the needle tip to face the opposite direction and extend out from it (Figure 1A). The puncture site was identified by creating indentations with Halsted mosquito forceps under direct laparoscopic visualization. The prepared catheter with 1-0 nylon thread was introduced through the abdominal wall into the abdominal cavity (Figures 2D,D’), and passed through the seromuscular layer of the stomach (Figure 1B). During this procedure, the stomach was grasped with fundus-grasping forceps to create a mucosal slip, ensuring that only the seromuscular layer was sutured (Figures 2E,E’). The catheter was simply withdrawn, leaving a loop of thread inside the abdominal cavity (Figures 1C, 2F,F’).

**Figure 3:**
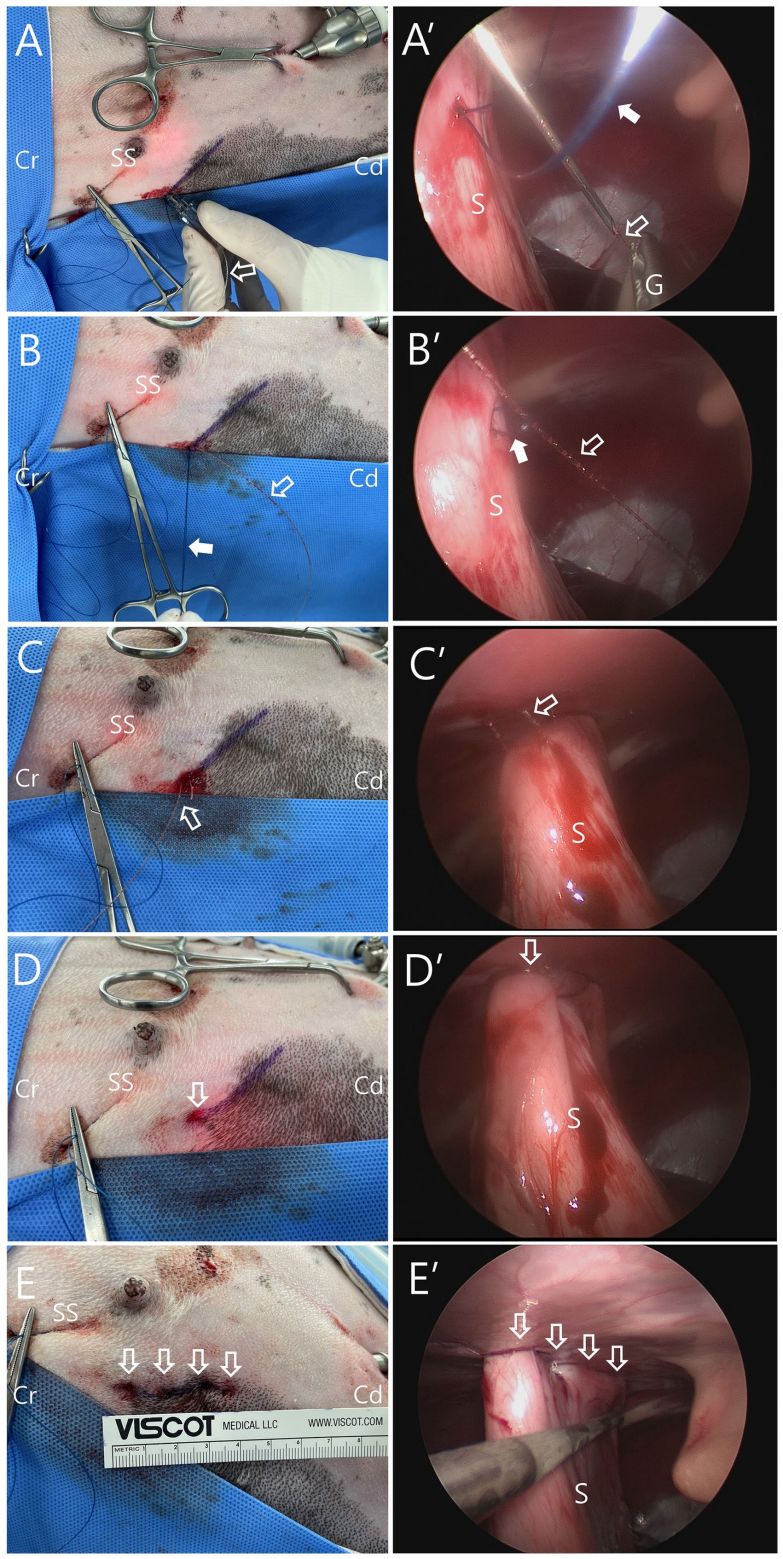
Intraoperative images of modified laparoscopic assisted percutaneous gastropexy (mLAPG) in a dog (X, X’: external and laparoscopic views at the same stage of the procedure). **(A)** The needle of the 16-gauge intravenous catheter with a 2–0 barbed suture (empty arrow) introduced into the previous skin puncture point for intraabdominal creation of nylon loop. **(A’)** The catheter with barbed suture (empty arrow) passing through the nylon (arrow) loop. The end of the barbed suture is held by grasping forceps (G). **(B,B′)** After catheter removal, the nylon (arrow) loop was pulled outward, bringing the barbed suture (empty arrow) caught in the nylon loop to the outside. **(C)** The nylon loop is completely pulled out, and both ends of the barbed suture (empty arrow) coming out through the same skin puncture point where the 16-guage catheter needle had previously introduced. **(C′)** The barbed suture (empty arrow) loop incorporating the seromuscular layer of the stomach. **(D,D′)** Both ends of barbed suture tied with a square knot (empty arrow). **(E,E’)** Completed gastropexy using four bites sutured with barbed suture (empty arrows). Cr, the cranial part of the patient; Cd, the caudal part of the patient; SS, stay suture; S, stomach.

As the second preparation, the unidirectional barbed suture (2-0 V-Loc 180, 45-cm barbed suture, Covidien, Dublin, Ireland), with its needle and welding loop removed, was passed through the 16G catheter by positioning the end of the suture entirely inside the catheter needle. Subsequently, the catheter was introduced into the abdominal cavity through the same skin puncture site used for the previous catheter with the nylon loop, while utilizing a distinct puncture site in the abdominal wall (Figures 1D, 3A). In the abdominal cavity, after the catheter needle passed through the nylon loop, the end of the barbed suture was advanced through the loop and held with laparoscopic grasping forceps (Figures 1E, 3A’). After the catheter was removed (Figure 1F), the nylon loop was pulled out of the abdominal cavity, bringing the barbed suture along with it (Figures 1G, 3B,B’). As a result, one end of the barbed suture passed through the seromuscular tract created by the first catheter insertion, and both ends of the barbed suture were brought out through the same skin puncture site (Figures 1H, 3C,C’). After a square knot was made, the suture was cut leaving a 2-mm tail, which was then buried under the skin (Figures 1I, 3D,D’). The same process was repeated three more times to create four bites of mLAPG (Figures 3E,E’). After removing the stay suture, the abdomen was deflated and the two portal sites were closed using a standard technique. The gastropexy site with 4 buried knots appeared cosmetically intact. The total gastropexy time (from port placement to port site closure) was 56 min, and the gastropexy suturing time (from the placement of the stomach stay suture to the final tied suture) was 29 min. The patient received tramadol 3 mg/kg IV TID (Tramadol HCl inj^®^, Shinpoong Pharm, Korea) for pain relief and cefazolin 22 mg/kg IV TID as antibiotic therapy, each administered for 3 days. On postoperative days (POD) 3 and 7, abdominal radiography and blood examinations (CBC, serum chemistry, electrolyte panel, and canine C-reactive protein (cCRP)) were performed, and no abnormalities were identified. Although the patient had nearly recovered by POD 7, discharge was delayed until POD 14 due to the owner’s circumstances.

**Figure 4:**
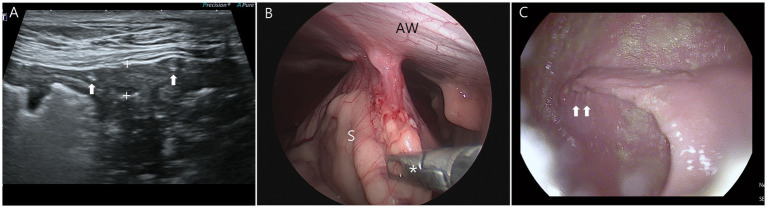
Follow-up images at 1 month after modified laparoscopic assisted percutaneous gastropexy (mLAPG) in a dog. **(A)** The hyperechoic structures (arrows) represent the 2–0 barbed suture material remaining within the muscularis layer of the stomach (calipers) as seen on ultrasonography. **(B)** The laparoscopic fundus-grasping forceps (asterisk) are pulling the stomach to assess adhesion formation and its tension under laparoscopic visualization. **(C)** A fold is visible at the gastropexy site (arrows), but no suture penetration is identified on gastroscopy. S, stomach; AW, abdominal wall.

At the 1-month postoperative follow-up, ultrasonography showed an intact gastropexy site without granulomas or seromas in the gastric wall or surrounding tissues (Figure 4A). No sliding motion between the stomach and the abdominal wall was observed during gastric motility or respiratory movements (Supplementary material 1). And the hyperechoic suture material (2-0 barbed suture) was identified on the gastropexy site. Laparoscopic and endoscopic evaluations were performed concurrently under the same anesthesia, following the protocol applied in the previous mLAPG procedure. Under laparoscopic evaluation, the pyloric antrum was retracted from the abdominal wall using laparoscopic grasping forceps with appropriate force (Figure 4B; Supplementary material 2). It subjectively demonstrated adequate adhesion between the abdominal wall and the pyloric antrum. Adjacent abdominal organs were not entrapped at the gastropexy site. On endoscopic evaluation, folds in the gastric wall at the gastropexy site were observed, and the absence of intraluminal suture penetration or abnormal gastric lesions was confirmed (Figure 4C). During a telephone interview, the owner reported that the dog had remained healthy and free of gastrointestinal symptoms throughout the 1-year postoperative period.

## 3 Discussion

The risk of GDV increases with age, possibly owing to the progressive stretching of the hepatogastric ligament over time (31–33). Therefore, patients are typically middle-aged or older dogs. GDV is more common in large breeds (weight > 23 kg) and tends to occur more frequently in purebred than in mixed-breed dogs (31, 32). Deep chest with a higher thoracic depth-to-width ratio represents another significant risk factor (31, 32, 34). Dogs with genetic predispositions such as a family history of GDV are also at higher risk (34). In this case, the patient was a 25-kg purebred Russo-European Laika with a thoracic depth-to-width ratio of 1.39, categorizing her as a deep-chested dog (depth-to-width ratio >1.25) (35). She was 5 years old, an age at which the risk of GDV tends to increase (33). Most importantly, her mother dog had died of GDV. These factors placed the patient in a high-risk group, constituting the primary indication for selecting this case for mLAPG.

In mLAPG, a self-anchoring barbed suture was used at the gastropexy site to induce permanent adhesion (22). The barbs of the self-anchoring barbed suture increase the contact area between the suture and the tissue, thereby enhancing load resistance and reducing the risk of gastropexy failure (36). To achieve permanent attachment, most conventional gastropexy techniques aim to promote healing between the incised seromuscular layer of the stomach and the transverse abdominis muscle (4, 20–22, 27). mLAPG in the present case caused no incision or abrasion to the seromuscular layer of the stomach or the transversus abdominis muscle. According to Deroy et al., each barb along the self-anchoring barbed suture securely engages the tissue, resists tissue pull-out (23). It maintains consistent tension and induces sufficient trauma to encourage fibrous adhesion, even without the need for an incision or abrasion.

A previous cadaveric study compared mLAPG and a simple continuous suture (TLG), both performed with barbed sutures (37). The tensile strength after mLAPG (35.86 ± 8.24 N) was significantly higher than that following TLG (24.04 ± 7.16 N), demonstrating that mLAPG provides greater mechanical strength. According to the study, it suggests that barbed sutures in mLAPG engage the abdominal wall in full thickness, which may apply a stronger force between the suture and the abdominal wall. This indicates that the mLAPG could provide a more durable and mechanically stable gastropexy, which further supports its potential as a reliable alternative for GDV prophylaxis.

Owing to absence of intracorporeal suturing, mLAPG is less technically challenging than TLG. In addition, because mLAPG does not require the use of a laparoscopic needle holder, it allows for a simpler instrumental setup. In a previous study by Mayhew and Brown, the average gastropexy suturing time (from the end of portal placement until the portals were ready to be removed) for incisional TLG using intracorporeal suturing was 48 min (range, 39–61 min) (19). mLAPG required 29 min for gastropexy suturing (from the placement of the stomach stay suture to the final tied suture). Considering that this was the first case in which mLAPG was performed in a clinical patient, the gastropexy suturing time is expected to decrease further with increased proficiency. Compared to conventional TLG, which requires three ports or a larger single-incision laparoscopic surgery port (4, 19, 22–24, 27), mLAPG uses only two 6-mm ports, thereby minimizing the risk of complications associated with port insertion.

As for postoperative follow-up of gastropexy site, ultrasonography is a noninvasive and repeatable technique that typically does not require sedation. The presence of the suture material in the muscular layer indicates that the submucosa and mucosa slipped away from the seromuscular layer during the mucosal slip. This allowed the suture bites to be securely incorporated into only the seromuscular layer, without penetrating the submucosa or mucosa (23, 38). Moreover, no sliding motion indicates that appropriate adhesion development was achieved using the mLAPG procedure (20, 23, 39, 40). However, the absence of the sliding motion cannot be used to assess the quality or strength of the gastropexy.

Laparoscopic follow-up allows for the subjective assessment of adhesions at the gastropexy site (16, 23, 24). Laparoscopic evaluation provided direct visualization of the gastropexy site, allowing gross assessment of adhesion formation and confirmation that no adjacent structures were entrapped. In addition, by retracting the adhered stomach from the abdominal wall using laparoscopic grasping forceps, the strength of adherence could be assessed and was found to be adequate.

In combination of laparoscopic evaluation, endoscopy was performed. The light of the laparoscope was directed close to the suture line at the gastropexy site within the abdominal cavity, allowing endoscopy within the stomach to accurately locate and evaluate the gastropexy site. To the best of our knowledge, there has been no reported case wherein endoscopy was used for postoperative evaluation after laparoscopic gastropexy. In one study, fistula formation around the suture material was reported as a long-term postoperative complication of laparoscopic gastropexy (23). Such fistulas may result from intraluminally placed gastropexy sutures. Endoscopy is the most definitive method for confirming the absence of intraluminal sutures after gastropexy. In the present case, no intraluminal sutures or gastric abnormalities were detected.

In this study, additional fibrous adhesions developed postoperatively in the live patient, which may have resulted in an even greater tensile strength than that measured in the cadaveric models (37). In the study by Spah et al., adhesion formation at 1-month postoperatively in incisional gastropexy using barbed sutures was confirmed through ultrasonography (4). In the present study, despite being a non-incisional gastropexy using barbed sutures, we confirmed that adhesion formation could occur at 1-month postoperatively. Ultrasonography revealed the absence of sliding motion, whereas laparoscopy confirmed stable adhesions, which were verified using laparoscopic forceps. These methods were validated in previous gastropexy studies (16, 23, 24, 39, 40). Therefore, mLAPG may be a reproducible and effective prophylactic gastropexy technique in dogs.

In dogs, prophylactic gastropexy is often performed concurrently with other procedures, including ovariectomy (OVE). In two-port laparoscopic OVE, the portal site placement is similar to that of mLAPG; therefore, these two procedures could be feasibly combined. Accordingly, performing mLAPG concurrently with OVE in female dogs may be considered a practical option. Although these combinations have not yet been attempted and potential challenges cannot be fully anticipated, it appears feasible to apply the technique to other procedures such as cryptorchidectomy or liver biopsy. However, depending on the case, the surgical approach may be limited or an additional port may be required.

Our study had some limitations. First, although a telephonic follow-up was conducted for 1 year, the postoperative follow-up data was limited to only 1 month owing to the owner’s difficulty in visiting the hospital. Second, as this report describes only a single case, further long-term studies involving more patients will be needed to evaluate the clinical efficacy of mLAPG in the prevention of GDV.

In conclusion, the mLAPG technique in the present report eliminates the need for intracorporeal suturing, making it technically less challenging. This incisionless method is less invasive compared to other laparoscopic gastropexy techniques, requiring only two 6-mm ports. The procedure was successfully performed in the patient, achieving secure adhesion without injury to the gastric wall or any gastropexy-related complications. Consequently, mLAPG can be recommended as a safe and effective approach for prophylactic gastropexy in dogs.

## Data Availability

The original contributions presented in the study are included in the article/Supplementary material, further inquiries can be directed to the corresponding author.
