# Correction: Case Report: Successful application of modified laparoscopic assisted percutaneous gastropexy in a dog using two 6-mm portal sites

**DOI:** 10.3389/fvets.2025.1765984

**Published:** 2026-01-13

**Authors:** Frontiers Production Office

**Affiliations:** Frontiers Media SA, Lausanne, Switzerland

**Keywords:** laparoscopy, prophylactic gastropexy, gastric dilatation and volvulus (GDV), laparoscopic gastropexy, barbed suture, minimal invasive surgery (MIS)

Author Seung Jun Yoon was omitted as an author in the published article. The correct author list reads:

Sohee Youn^1^, Ho Hyun Kwak^1, 2^, Seung Jun Yoon^1^, Junhyung Kim^1^ and Heung Myong Woo^1*^

^1^Department of Veterinary Surgery, College of Veterinary Medicine, Institute of Veterinary Science, Kangwon National University, Chuncheon, Republic of Korea

^2^Department of Companion Animal Industry, College of Natural and Life Sciences, Daegu University, Gyeongsan, Republic of Korea

The Author Contributions Statement has been corrected to read:

SoY: Data curation, Investigation, Writing – original draft. HK: Writing – review & editing, Visualization. SeY: Writing – original draft, Writing – review & editing, Methodology, Conceptualization, Investigation. JK: Writing – review & editing. HW: Writing – review & editing, Supervision.

Affiliation “Department of Veterinary Surgery, College of Veterinary Medicine, Institute of Veterinary Science, Kangwon National University, Chuncheon, Republic of Korea” was erroneously given as “Department of Veterinary Surgery, College of Veterinary Medicine, Kangwon National University, Chuncheon, Republic of Korea.”

The original version of this article has been updated.

